# Evaluation of the measles case-based surveillance system in Kwekwe city, 2017-2020: a descriptive cross-sectional study

**DOI:** 10.11604/pamj.2022.42.113.31373

**Published:** 2022-06-10

**Authors:** Nyashadzashe Cosmas Makova, Mary Muchekeza, Emmanuel Govha, Tsitsi Patience Juru, Notion Tafara Gombe, Maurice Omondi, Mufuta Tshimanga

**Affiliations:** 1 Department of Primary Health Care Sciences, Global and Public Health Unit, University of Zimbabwe, Harare, Zimbabwe,; 2 Kwekwe City Council Department of Health, Kwekwe, Zimbabwe,; 3 African Field Epidemiology Network, Harare, Zimbabwe,; 4 African Field Epidemiology Network, Nairobi, Kenya

**Keywords:** Measles, surveillance, Kwekwe city, Zimbabwe

## Abstract

**Introduction:**

in 2011, WHO African region set a target for elimination of measles by 2020. During period 2017-2020, Kwekwe city, with an estimated population of 117,116, detected one case of suspected measles. This was against a target of 2 cases per year. We evaluated the system to establish why it was failing to detect at least 2 cases per year.

**Methods:**

we conducted a descriptive cross-sectional study using the Centre for Disease Control (CDC) Updated Guidelines. Nineteen health facilities were selected and fifty-seven health workers were randomly recruited. An interviewer-administered questionnaire and checklists were used to collect data. We generated frequencies, proportions, and means.

**Results:**

the mean years in service was 22.8 years (SD=12.6). Thirty (52.6%) respondents had fair knowledge. Fourteen (73.7%) of the nineteen respondents who had ever completed case investigation forms took between 10-20 minutes to complete. Only two (10.5%) of the nineteen facilities had case investigation forms. The majority of the respondents 54 (93%) were willing to continue participating in the measles Community Base Surveillance System (CBSS). None of the health facilities had used the system to inform decision-making. Reasons highlighted for poor suspected measles case detection included lack of health worker training 28/57 (49.1%).

**Conclusion:**

despite the high age in service, knowledge of the surveillance system was mostly fair. The system was found to be simple, not stable and not useful. The main reason for the system failure was lack of health worker training. We recommend retraining on Integrated Disease Surveillance and Response (IDSR) and case investigation forms distribution.

## Introduction

Surveillance is the ongoing systematic collection, analysis, and interpretation of health data essential to the planning, implementation and evaluation of public health practice [1-3]. Measles is one of the vaccine preventable infectious diseases still causing mortality and morbidity in developing countries [4, 5]. Measles is highly contagious, and vaccine preventable [6, 7]. Infection with the virus can cause serious infections that can lead to complications, and death [8-10]. Transmission of the virus is through person-to-person spread, primarily through aerosols [11]. Infected individuals typically present with a maculopapular rash, fever, cough, coryza, and conjunctivitis [12]. Measles is usually self-limiting, and its clinical presentation may be the same with other viral infections [11].

Measles elimination is the absence of endemic measles virus transmission in a well-defined geographic area for at least 12 months in the presence of a well-performing surveillance system [13, 14]. Following the elimination of measles in the Americas, the other five regions of WHO adopted the measles elimination targets to be reached by the end of 2020 [11]. The elimination targets are measles incidence of less than 1 case per million population at national level, at least 95% measles immunization coverage at national level and in all districts, minimum 95% coverage in all measles supplemental immunization activities (SIAs), at least 80% of districts investigating one or more suspected measles cases within a year, and a non-measles febrile rash illness rate of at least 2 per 100 000 population at national level. In 2011, WHO African region set a target for elimination of measles and to achieve this, three main strategies to be employed were achieving high routine immunization, high SIAs and case-based surveillance system with improved case management [15].

As progress towards measles elimination is made, surveillance system should be more sensitive [7, 13]. To achieve this, all cases with fever, and rash should be identified, and investigated [8]. During the period 2017 to 2019, measles incidence increased in all the WHO regions. In 2018, only five countries in the World Health Organization (WHO) AFRO region accounted for approximately half of all measles cases reported globally [16]. In 2019, all the 194 WHO member states conducted measles surveillance [17]. However, measles surveillance was noted to be weak. By the end of 2019, none of the WHO region had achieved and maintained measles elimination targets [17]. Furthermore, the coronavirus pandemic presented programmatic challenges leading to more children being missed for vaccinations, and poorer surveillance [17].

Zimbabwe is now in the measles pre-elimination phase, and has to meet all targets by 2020 [15]. In 2018, Zimbabwe reported only one measles case to WHO [18] and four cases of measles in 2019. This was a significant increase as the country was reporting only one case annually for the period 2016 to 2018 [18]. Measles case-based surveillance is integrated with other vaccine preventable diseases like acute flaccid paralysis (AFP) [8]. Suspected cases should be identified, notified within seven days of rash onset, and fully investigated within 48 hours of notification. A serological sample should be collected within 30 days of the onset of the rash [5]. In Zimbabwe, surveillance data for suspected measles cases is routinely collected at all levels of the health delivery system using the measles case surveillance form. Primary health facilities send the data to the district, and the data is transmitted to provincial and national level.

There has not been a recorded suspected case of measles in Kwekwe city, Zimbabwe from 2017 against a target of identifying two suspected cases per year. Failure to detect possible imported or emergence cases of measles can result in the disease spreading, invariably leading to an increase in morbidity and mortality from measles infection. We evaluated the measles case-based surveillance system to assess knowledge of health workers, its attributes and to determine the reasons for failure to meet the suspected cases target.

## Methods

**Study design:** we conducted a descriptive cross-sectional study in Kwekwe city using the 2001 updated Center for Disease Control (CDC) guidelines for surveillance system evaluation [2]. The CDC guidelines are recommendations from the guidelines working group which provide updated guidelines for evaluating surveillance systems based on CDC's Framework for Program Evaluation in Public Health. The guidelines describe tasks and activities that can be applied to public health surveillance systems [2]. A descriptive cross-sectional study is a study in which the condition under measure and potentially related factors are measured at a specific point in time for a defined population. We conducted a descriptive cross-sectional study because it is relatively fast and inexpensive and would best answer the research question.

**Study setting:** we conducted the study in health facilities in Kwekwe city. According to the Zimbabwe National Statistics Agency (ZIMSTATS) Midlands Province District Population Projections report of 2020, the current population of Kwekwe city is approximately 117,116. There are five council clinics namely Al Davies, Amaveni, Mbizo 1, Mbizo 11, and Mbizo 16. There are 19 private health facilities, constituting 79.2% of the health facilities. All the health facilities in Kwekwe city are involved in the measles case-based surveillance system.

**Study participants and sampling:** the health care workers who include registered midwives, registered general nurses, doctors, records consisting of outpatients´ department record (T12 and Integrated Management of Childhood and Infant registers) and measles case notification form were the study population. The assistant director for health from the nursing side and environment side were recruited as key informants. We used the Dobson formula


$$n=\frac{Z{\left(\frac{\alpha }{2}\right)}^{2}P(1-P)}{\delta^{2}}$$

Based on a study done in Gokwe North district by Makoni where the reported reason for failure to detect cases was communities not reporting cases (82%), at 95% confidence level, z=1.96 and δ=0.1(10% precision), and a non-response rate of 10% [19]. We calculated a minimum sample size of 63 health care workers. We purposively selected four of the five council clinics into the study because they are the high volume facilities. We randomly sampled 15 of the 19 registered private health facilities in Kwekwe city. The private health facilities in Kwekwe city were listed and assigned numbers. The Excel “=RAND0” function was used to randomly select the 15 health facilities. We randomly selected 33 health care workers at the four council clinics and 24 health workers from the 15 enrolled private sector.

**Data collection:** we used a pre-tested semi structured interviewer-administered questionnaire to collect data from health care workers on measles case-based surveillance system. The questionnaire had four main sections which were the demographic characteristics, knowledge assessment, reasons for failure and attributes such as simplicity, stability, acceptability and sensitivity. The demographic characteristics included gender, age, number of years in service and designation. We pretested the questionnaire by interviewing health care workers at Redcliff clinic where the setup was similar to that in kwekwe city. We went to the selected facilities and conducted interviews using the questionnaires. Furthermore, we reviewed records using a checklist to assess some system attributes. An interviewer guide was used to collect data from key informants and three main sections, demographic characteristics, operation of the measles surveillance system and reasons for failure.

We assessed health care workers´ awareness and understanding of the components of the measles case based surveillance system by asking participants to answer questions regarding measles case based surveillance. We used an interviewer-administered questionnaire and asked six questions to assess knowledge levels of health workers. We defined variables and attributes based on the Centres for Disease Control and Prevention (CDC) Updated Guidelines for Evaluating Public Health Surveillance Systems. Simplicity was defined as the ease of operation of the surveillance system. We assessed simplicity by finding out whether implementers of the system had ever completed a case notification form and the amount of time they took to complete the form. We defined acceptability as the willingness of persons and organizations to participate in the surveillance system. Likewise, we used the interviewer-administered questionnaire to ascertain willingness to participate in the system. We objectively assessed acceptability by checking the completeness of measles case notification forms.

Sensitivity referred to the proportion of suspected measles cases detected by the surveillance system. We assessed sensitivity by calculating the proportion of suspected cases of measles reported by the surveillance system with those that were in the health records that met the definition but were not reported by the surveillance system. Timeliness reflects the speed between steps in a public health surveillance system. We assessed timeliness by checking whether cases were investigated within 48 hours following notification, whether serum samples were sent to the virology laboratory within three days and whether results were received from the virology laboratory to the health facility within the stipulated seven days. We used checklists to assess the availability of necessary resources such as specimen collection tubes, transport, telephones and stationery.

**Data analysis:** we used Epi Info 7.2.4.0™ (CDC, 2020) statistical package to capture and analyse data from questionnaires. Frequencies, proportions, means and corresponding standard deviations were calculated using the same software. We analysed qualitative data from checklists and key informants manually. We rated health care workers´ knowledge of the surveillance system using a three-point score. Six questions were asked and those who answered 1-2 questions correctly regarded as having poor knowledge, 3-4 fair and 5-6 good. The grading of the score was based on a study by Saravoye *et al*., 2015 in evaluating the Acute Flaccid Paralysis (AFP) surveillance system in Zvimba District, Mashonaland West Province where knowledge of respondents on AFP surveillance was aggregated on a scale of 1-6 and graded as follows: poor (<3); average (3-4) and good (5-6).

**Ethical considerations:** we obtained permission to conduct the study from the director of health services of Kwekwe city and the health studies office (HSO). We obtained informed written consent from all the interviewees and we assured them of confidentiality. To ensure confidentiality, we stored the completed forms in a locked cabinet and electronic records were stored in a password protected compute. All information that would identify a participant was not included. Since data collection took place during the COVID-19 pandemic, the essential precautions were strictly adhered to by ensuring social distancing between the interviewer and study participants, hand hygiene and wearing masks that adequately covered the nose and mouth.

## Results

We visited all the 19 health facilities. We recruited 57 participants, of these, 63.2% (n=36) were females. The majority of the respondents (35.1%) were registered general nurses. The majority of the respondents (29.8%) had more than 30 years in service, and the mean years in service was 22.8 years (SD=12.6) (Table 1).

**Table 1:** demographic characteristics of health workers in Kwekwe city, Zimbabwe, 2020

Characteristic	Category	Frequency n=57(%)
**Facility type**	Council	31 (54.4)
	Private	26 (45.6)
**Designation**	Registered general nurse	20 (35.1)
	Midwife	19 (33.3)
	Sister in charge	4 (7.0)
	Medical Doctor	14 (24.6)
**Sex**	Male	21 (36.8)
	Female	36 (63.2)
**Years in service**	Less than 10	13 (22.8)
	10 to 20	12 (21.1)
	21 to 30	15 (26.3)
	Greater than 30	17 (29.8)
**Mean years in service**	22.8 (SD=12.6)	

**Health worker knowledge on the measles case-based surveillance system:** all the study participants 57 (100.0%) knew the correct case definition of a measles case. Fifty (87.7%) of the respondents knew that a blood sample was collected for investigation. Ten (17.5%) of the respondents knew the number of investigation forms completed per case. Sixteen (28.1%) had good knowledge of the measles case-based surveillance system (Table 2).

**Table 2:** knowledge levels of health workers in the measles case-based surveillance system, Kwekwe city, Zimbabwe, 2020

Variable	n = 57	Percentage
Case definition	57	100.0
Type of sample collected for investigation	50	87.7
Phase in which Zimbabwe is on measles surveillance	46	80.7
Time limit for sample to reach laboratory	19	33.3
Time limit to investigate suspected measles case	16	28.1
Number of investigation form copies to be filled	10	17.5
**Summary of knowledge level**		
Good (5-6 Correct)	16	28.1
Average (3-4 Correct)	30	52.6
Poor (0-2 Correct)	11	19.3

### Measles case-based surveillance system attributes

**Simplicity:** nineteen (33.3%) of the study participants had ever completed a measles case notification form. Four of 19 reported taking less than 10 minutes to complete the forms. Ten of 19 reported taking between 10 and 20 minutes. Five of 19 respondents reported taking more than 20 minutes to complete the forms. Two health workers were each timed completing the five case investigation forms and the average time to complete was less than 12 minutes highlighting relative simplicity in filling the reporting forms.

**Acceptability:** the majority of the respondents 53 (93.0%) reported they were responsible for filling the notification forms. Fifty-four (93.0%) of the respondents were willing to continue participating in the system. The only form that was identified was incompletely filled and results had not been received.

**Sensitivity:** Kwekwe city´s target for the measles case-based surveillance were two cases per 100 000 population. The system did not pick any case from 2017 to 2019. The system picked one case in 2020 and the sensitivity was 50%. There was no result for the case. Nine cases meeting the case definition of measles were missed as reviewed in the outpatients and integrated management of neonatal and childhood illness (IMNCI) registers.

**Stability:** ten (17.5%) of the participants had received training on specimen collection and completion of forms. Two (10.5%) of the 19 health facilities had case notification forms which were inadequate. Fifteen (78.9%) health facilities had collection tubes and six (31.6%) had Expanded Programme on Immunization (EPI) Guidelines available. All the health facilities had a functional cell phone and transport available. Five (26.3%) facilities had measles case definition displayed (Table 3).

**Table 3:** stability of measles case-based surveillance system in Kwekwe city, Zimbabwe, 2017-2020

Variable	n = 19	Percentage
Health facilities with collection tubes	15	78.9
Case definition displayed	5	26.3
Measles case investigation forms available	2	10.5
EPI Guidelines available	6	31.6
Cell phone or landline available	19	100.0
Transport available	19	100.0

**Timeliness:** specimen collection was done at the facility level and investigation were done at the national level. The identified case was reported more than 7 days after the onset of the rash. It was investigated within 48 hours, there was no documentation of the results reaching the lab and results were not received within seven days.

**Reasons for the underperformance of the measles case-based surveillance system:** reasons highlighted for poor suspected measles case detection included lack of training on the measles case-based surveillance system 28/57 (49.1%), poor active case finding 9/57 (15.8%) and lack of community awareness 17/57 (29.8%). Some other reasons noted to contribute to failure of the system were usage of contract nurses, poor inclusion of private facilities in meetings and workloads that were high (Table 4).

**Table 4:** reasons for the underperformance of the measles case-base surveillance system in Kwekwe city, Zimbabwe, 2017-2020

Variable	n = 57	Percentage
Lack of training on the surveillance system	28	49.1
Lack of community awareness	17	29.8
Poor health care worker knowledge on measles surveillance	13	22.8
Lack of active case finding	9	15.8
Unavailability of measles investigation forms	7	12.3
Other	7	12.3

## Discussion

We evaluated the measles case-based surveillance system to assess knowledge of health workers, its attributes and determined the reasons for failure to meet the suspected cases target. We found that knowledge was mostly fair, the system was simple, and however it lacked sensitivity, timeliness, acceptability and stability. Lack of training contributed to failure to detect suspected cases of measles.

The majority of the study participants had at least fair knowledge of the measles case-based surveillance system when measured with the three-point scale. This was despite the mean age in service which was relatively high. With increase in age and in service experience, it is likely to increase and this will likely increase knowledge and acceptability of the system as was found by Ng´etich in Kenya [21]. The fair knowledge could be attributed to lack of training on the measles case-based surveillance system. Having good knowledge of the measles case-based surveillance system is of paramount importance in the effectiveness and efficiency of the system and will also help detect outbreaks. The findings were contrary to the findings in Masvingo district where the majority of the participants had excellent knowledge of the system [20].

The measles surveillance system was found to be simple as the majority of participants who had ever completed a notification form took less than 20 minutes. The system could also have been simple because of the experience level of the healthcare workers due to the high mean age in service. When a surveillance system is simple, this will likely increase acceptability of the system and will in turn meet its objectives. Debela in Ethiopia found the measles surveillance system to be simple. This was because the amount of time spent operating the surveillance system was less than 20 minutes [21].

We found that the majority of the participants stated that it was their duty to complete the notification forms and also it was necessary to complete notification forms and notify cases. However, the measles case-based surveillance system was found to be not acceptable as the system lacked timeliness. This could be attributed to the lack of training on the measles case-based surveillance system and also the employment of a lot of locum nurses who also lack the motivation to work. Moreover, some nurses may not want the extra responsibility associated with notifying a case as they are locum nurses. A surveillance system that is not acceptable will not be used by the different stakeholders and will not meet its objectives. The findings were not consistent with those by Ameh who concluded that the surveillance system was acceptable to all stakeholders and operators of the surveillance system [22].

We also found that the measles case-based surveillance system lacked sensitivity in Kwekwe city. The system failed to meet the recommended targets and also failed to detect suspected cases of measles nonetheless, a review of the outpatients and Integrated Management of Neonatal and Childhood Illness registers had cases that fit the definition. The reduced sensitivity could be attributed to the employment of locum nurses who may have limited accountability when compared to permanent nurses. Such low sensitivity of a vital surveillance system like measles surveillance is detrimental to public health and this might be attributed to the lack of training in the surveillance system. This may result in surveillance system being not able to detect outbreaks. The findings were in keeping with those in River State, Nigeria, where the sensitivity never met the recommended target throughout the entire period and seemed to decline even further from 2015 [23].

Measles case-based surveillance system for Kwekwe city lacked timeliness. This could be attributed to lack of community knowledge because of reduced community awareness. Lack of community knowledge on measles was cited as a reason for failure of the system by approximately a third of the study participants and also the city has no community nurse. Community nurse´s role involves much more preventative, community-centred activities such as health awareness. A system that lacks timeliness will respond late to outbreaks. Aworabhi-Oki concluded that Measles case based surveillance system for Bayelsa State, Nigeria lacked timeliness in transportation of specimens to the laboratory and low feedback of laboratory results [24]. Contrary to the study findings, Choto found out that timeliness was improving in the measles case based surveillance system in Zimbabwe [25] and also Ameh in Kaduna State, Nigeria found the system to be timely as 83% of reports were above the WHO target of 80% [22].

In Kwekwe city, the measles case-based surveillance system was found to be unstable. The stability of the system was affected by the unavailability of resources like case notification forms. Furthermore, the pressure of the coronavirus pandemic on the health systems could have further affected the stability. None of the private facilities in kwekwe city had notification forms and less than a quarter of the private facilities had EPI guidelines. This could be attributed to poor integration of the private facilities into the system or the private sector perceive the system as non-profitable to them as it does not directly generate revenue for them. Findings were consistent with those made by Makoni in Gokwe North, Midlands Province, where stability of the AFP surveillance system was affected by shortages of resources such as case notification forms. Furthermore, Saravoye in Zvimba District, Mashonaland West Province found that only one of the seven private health facilities in the study had AFP case notification forms [19, 26].

We found that lack of training contributed to the failure of the surveillance system in Kwekwe city. When health care workers lack critical knowledge on the surveillance system, it will not perform as expected. The Integrated Disease Surveillance and Response (IDSR) strategy is a comprehensive approach adopted by the African Regional Office of the World Health Organization (WHO/AFRO) in 1998 to improve disease surveillance in the region. It provides a framework for strengthening surveillance, response and laboratory core required by the revised International Health Regulation [IHR (2005)] and health workers are being trained on IDSR. To improve surveillance of the measles case-based surveillance, there is great need to train health care workers. It was found out that training improved the surveillance system in Yobe State, Nigeria [27]. In Uganda, healthcare workers cited improved completeness and timeliness of reporting, case detection and data analysis and better response to disease outbreaks as key achievements after the training [28].

**Limitations of the study:** we only reviewed one case notification form and this could have affected the assessment of other system attributes that include data quality and completeness. However, the limitations do not limit the interpretation of our findings on the performance of the measles case-based surveillance system in Kwekwe city.

## Conclusion

Despite the high mean age in service, the knowledge on the measles case based system was mostly fair. The main reasons for poor performance of the measles surveillance system were lack of training and lack of community awareness. The measles case-based surveillance system was found to be simple, not acceptable, untimely, not sensitive and unstable. We recommend integration with the private sector to improve the performance of the system in Kwekwe city. We further recommend awareness campaigns and health education programs in communities, employment of a community nurse and Continuing Medical Education (CME) activities on surveillance systems. Retraining and updates on Integrated Disease Surveillance and Response (IDSR) should be offered to health care workers. We photocopied and distributed measles case definitions to five private health facilities. Education on measles case based surveillance was given to health personnel during interviews.

### What is known about this topic

- Measles is targeted for eradication and surveillance systems should be more sensitive to achieve the target;
- Implementation of surveillance systems in developing countries is challenged by many factors.

### What this study adds

- This study found that poor integration with the private facilities threatens the measles case-based surveillance system;
- This study showed that a surveillance system that is not acceptable, untimely, not sensitive and unstable would not attain its intended objectives.
